# Correlation of ultra-high field MRI with histopathology for evaluation of rectal cancer heterogeneity

**DOI:** 10.1038/s41598-019-45450-2

**Published:** 2019-06-27

**Authors:** Trang T. Pham, Timothy Stait-Gardner, Cheok Soon Lee, Michael Barton, Petra L. Graham, Gary Liney, Karen Wong, William S. Price

**Affiliations:** 1 Department of Radiation Oncology, Liverpool Cancer Therapy Centre, Liverpool Hospital, Sydney, Australia; 2 South Western Sydney Clinical School, Faculty of Medicine, University of New South Wales, Sydney, Australia; 3 Ingham Institute for Applied Medical Research, Sydney, Australia; 4 Nanoscale Organisation and Dynamics Group, Western Sydney University, Sydney, Australia; 5 School of Medicine, Western Sydney University, Sydney, Australia; 6 Department of Anatomical Pathology, Liverpool Hospital, Sydney, Australia; 7 Centre for Economic Impacts of Genomic Medicine (GenIMPACT), Macquarie Business School, Macquarie University, Sydney, Australia

**Keywords:** Cancer imaging, Rectal cancer

## Abstract

Current clinical MRI techniques in rectal cancer have limited ability to examine cancer stroma. The differentiation of tumour from desmoplasia or fibrous tissue remains a challenge. Standard MRI cannot differentiate stage T1 from T2 (invasion of muscularis propria) tumours. Diffusion tensor imaging (DTI) can probe tissue structure and organisation (anisotropy). The purpose of this study was to examine DTI-MRI derived imaging markers of rectal cancer stromal heterogeneity and tumour extent *ex vivo*. DTI-MRI at ultra-high magnetic field (11.7 tesla) was used to examine the stromal microstructure of malignant and normal rectal tissue *ex vivo*, and the findings were correlated with histopathology. Images obtained from DTI-MRI (A0, apparent diffusion coefficient and fractional anisotropy (FA)) were used to probe rectal cancer stromal heterogeneity. FA provided the best discrimination between cancer and desmoplasia, fibrous tissue and muscularis propria. Cancer had relatively isotropic diffusion (mean FA 0.14), whereas desmoplasia (FA 0.31) and fibrous tissue (FA 0.34) had anisotropic diffusion with significantly higher FA than cancer (p < 0.001). Tumour was distinguished from muscularis propria (FA 0.61) which was highly anisotropic with higher FA than cancer (p < 0.001). This study showed that DTI-MRI can assist in more accurately defining tumour extent in rectal cancer.

## Introduction

Standardised surgical technique with total mesorectal excision, and multimodality treatment strategies with neoadjuvant chemoradiotherapy have led to improved survival in rectal cancer^1–3^. In selected patients with early rectal cancer, local excision may be an appropriate treatment option. Accurate pre-operative staging in rectal cancer is therefore of paramount importance in the selection of optimal surgical technique, and stratification of patients into those who can undergo surgery alone or those who would benefit from neoadjuvant chemoradiotherapy. Current clinical magnetic resonance imaging (MRI) techniques using *T*_2_-weighted imaging have an established role in the primary staging of rectal cancer, however, there are limitations with their use. Firstly, *T*_2_-weighted MRI is unable to accurately differentiate stage T1 (tumour invades submucosa) from stage T2 (tumour invades muscularis propria) tumours^4,5^, and endoscopic ultrasound is currently the recommended imaging modality of choice by the European Society of Gastrointestinal and Abdominal Radiology (ESGAR) to differentiate between T1 and T2 tumours if local resection is being considered^4^. Secondly, *T*_2_-weighted MRI has limited ability to examine the rectal cancer stromal microenvironment, and differentiation of tumour from desmoplastic reaction or fibrous tissue can be challenging. Stranding into the mesorectal fat is an equivocal sign that may indicate either a T2 tumour with desmoplasia or a T3 (tumour invades through the muscularis propria into perirectal tissue) tumour with tumoural strands^4^. Functional MRI has the capability to non-invasively characterise tumour heterogeneity and its role in more accurate staging of rectal cancer should be further explored.

Tumour stroma evolves during cancer progression and is associated with increased extracellular matrix. The increased deposition of extracellular matrix (fibrosis) in tumours is known as desmoplasia, and characteristic of many advanced cancers^6,7^. Histopathologic studies in colorectal cancer have identified changes in the tumour stroma, including increased collagen fibril stiffness and anisotropy, compared to normal healthy mucosa^8^. Fibril anisotropy (degree of organisation) may serve as a novel predictive stromal biomarker for cancer invasion and assist in more accurate staging in rectal cancer. Since the fibres cause anisotropic restriction of the diffusion of tissue water, diffusion tensor imaging (DTI) MRI may provide a non-invasive method for characterising such fibril anisotropy. DTI-MRI, which provides a rotationally invariant description of tissue diffusion for each voxel in the image, has the potential to supply additional information about the tumour stromal microenvironment. DTI-MRI can probe stromal microstructure and organisation (anisotropy). The apparent diffusion coefficient (ADC) and fractional anisotropy (FA) for each voxel can be calculated from the diffusion tensor measured for each voxel. Viable tumours restrict cellularity and water movement, resulting in a low ADC. FA may be useful for more accurate tumour delineation and detection of tumour invasion into tissues with highly structured organisation^9^. Most tumours typically exhibit relatively isotropic diffusion, whereas organised tissues exhibit anisotropic diffusion. To our knowledge, the potential clinical utility of DTI-MRI in rectal cancer has not yet been investigated in the *ex vivo* or clinical settings.

We hypothesise that DTI-MRI can assess stromal heterogeneity in rectal cancer, thereby allowing for more accurate delineation of rectal cancer extent and staging. This paper examines the potential of DTI-MRI to characterise fibril anisotropy as a probe of the stromal microenvironment and tumour extent. This proof of principle study was performed on an ultra-high field MRI at 11.7 tesla. The advantage of ultra-high field MRI is that the microscopic spatial (i.e., voxel) resolution (200 µm) can be used to reveal cancer microstructure *ex vivo*. This allows for direct correlation with ‘ground-truth’ histopathology analysis of the same tissue specimen.

The purpose of this exploratory study was to examine DTI-MRI derived imaging markers of rectal cancer stromal heterogeneity and tumour extent, *ex vivo*. Specifically, the stromal ultrastructure of rectal cancer and adjacent normal rectum was examined by high field strength DTI-MRI at 11.7 tesla *ex vivo*, and the MRI findings were correlated with histopathology.

## Methods

### Patients and biobank tissue collection

Patients with biopsy proven rectal cancer undergoing rectal surgery alone were eligible for this study. All patients who participated in this study provided written informed consent. All study protocols were approved by the institutional ethics committee South Western Sydney Local Health District (SWSLHD) Human Research Ethics Committee (HREC) (Approval numbers: HREC/14/LPOOL/152, HREC/14/LPOOL/370, Local Project Number 14/209, SSA/14/LPOOL/371, SSA/14/LPOOL/372, H10843). All study methods were carried out in accordance with SWSLHD HREC approved study protocols and SWSLHD Centre for Oncology Education Research Translation (CONCERT) Biobank procedures^10^.

Tissue was collected from the surgical specimens of rectal cancer patients. Two fresh tissue specimens were collected from each patient’s surgical specimen: (i) full thickness (containing both mucosal and serosal surface) rectal cancer with peri-lesional adjacent normal rectum, and (ii) full thickness adjacent normal rectum tissue 5–10 cm away from cancer. The specimens collected were up to 1.5 cm wide and 3 cm long, depending on the amount of tissue available for Biobank collection. Collected specimens were immediately fixed in 10% formalin for 24–48 hours. Tissue specimens were subsequently embedded in 1% agarose (1 g agarose in 100 ml distilled water) containing 2 mM gadopentetate dimeglumine (0.4 ml of Bayer Magnevist 0.5 M) for MR imaging. Care was taken to avoid tissue folding within the agarose gel. The orientation of mucosal and serosal surface in the MRI vial was marked. Photos were taken of gross specimens to document specimen orientation and aid in subsequent MRI – histopathology correlation.

### Magnetic resonance imaging

All rectal cancer and normal rectum tissue specimens were scanned on the Bruker Avance II 500 MHz (11.7 tesla) wide bore MRI spectrometer at the Western Sydney University Biomedical Magnetic Resonance Facility. The MRI vials were placed in the MRI bore with the specimen mucosal surface orientated to the left, and serosal surface orientated to the right of the MRI bore. An initial 3-dimensional MRI with 100 μm voxels (using Bruker’s TurboRARE-3D method with RARE factor 2, repetition time 300 ms, echo time 10.34 ms, 90° excitation pulse and 4 averages) was acquired for some specimens to facilitate anatomical registration of MR images with histopathology until it was determined that the A0 data from the DTI scan was equally suitable for this task. Three-dimensional spin-echo DTI were acquired with isotropic voxel resolution of 200 μm with *b*-values 200, 800, and 3200 s/mm^2^. The FOV was 27 × 27 mm in the axial plane and typically between 12 and 16 mm longitudinally depending on the length of the specimen. The echo time was 26 ms and repetition time was 900 ms. Eight diffusion directions were acquired with 3 diffusion experiments per direction. One A0 (i.e., *b* = 0) image was acquired. The diffusion gradient separation was 15 ms, and diffusion gradient duration was 5 ms. No image acceleration such as echo-planar imaging (EPI) was used leading to relatively long experimental times between 41 and 67 hours.

### Histopathology examination

Following *ex vivo* MR imaging, the rectal cancer and normal rectum tissue specimens were histologically examined by light microscopy. Specimens were sectioned axially from mucosal surface to serosal surface, as per conventional histopathology sectioning for diagnostic purposes. The slice thickness was 90 µm. Sections were mounted onto slides and stained with haematoxylin and eosin (H&E) stain, masson trichome stain and elastic Van Gieson (eVG) stain. Unmarked histopathology slides were scanned with an Aperio ScanScope Model CS digital scanner at 40× resolution. Histopathology slides could be viewed at any resolution up to a maximum resolution of 40× (0.25 µm/pixel) using the zoom window.

H&E stains were used to assess cancer and normal rectum ultrastructure. The masson trichome and eVG stains were used to analyse the extracellular matrix ultrastructure, and identify regions of desmoplasia and fibrosis. Histopathology assessment was undertaken by a pathologist with sub-specialisation in gastrointestinal pathology. Regions of interest were annotated on the digital histopathology images, viewed via Aperio ImageScope (version 12.3.2.9013). The following regions of interest were annotated on rectal cancer digital histopathology images: (a) cancer (b) cancer-associated desmoplasia (c) fibrous tissue and (d) non-malignant rectum (if present). The histologic sub-type and depth of carcinoma invasion into the rectal wall was also assessed. The following regions of interest were annotated on normal rectum digital histology images: (a) mucosa (b) submucosa (c) muscularis propria and (d) serosa. Any additional regions of interest that were identified in the histopathology specimen, such as granulation tissue, were also annotated.

### MRI - Histopathology correlation and co-registration

A pathologist sub-specialising in lower gastrointestinal malignancy, and a radiation oncologist with MRI expertise sub-specialising in lower gastrointestinal malignancy worked together to match the MRI with histopathology. Either the 3-dimensional MRI TurboRARE sequence or the A0 data from the DTI scan was used to select the MRI slice that visually matched with histopathology. As the MRI voxels acquired were isotropic, the MRI data could, through software processing, be visualised along arbitrary axes to ensure a good match with histopathology. The documented orientation of mucosal and serosal surfaces in the MRI vial, gross specimen images, and histopathology slicing direction (axial from mucosal to serosal surface) were used to indicate virtual slicing direction on MRI TurboRARE images to match the histopathology slice. The MRI TurboRARE dataset was aligned with the DTI-MRI dataset, allowing for automatic translation of the selected MRI TurboRARE slice to the corresponding DTI-MRI slice for analysis. Anatomic landmarks that were identifiable on both the MRI TurboRARE sequence and H&E slides were used to correlate MRI with histopathology. These landmarks included the specimen contour along the outer and inner rectal wall (including peaks and curvatures along the edges), mucosal surface, muscularis propria, serosal surface, and blood. A minimum of 7 landmarks were identified for MRI – histopathology correlation. Tissue regions, including tumour, fibrous tissue, desmoplasia, and rectal wall layers, identifiable on both modalities were contoured on corresponding DTI-MRI and histopathology images for subsequent evaluation of co-registration.

The DTI-MRI slice for analysis and annotated histopathology image were then co-registered to validate the visual match. A rigid co-registration method developed by Reynolds *et al*.^11^ was used to fuse the DTI-MRI slice with annotated histopathology using the MATLAB Image Processing Toolbox software R2018a version 16.4 (MathWorks, Natick, United States). This co-registration method was previously quantitatively validated by Reynolds *et al*. and found to have a mean distance of 0.57 mm between control points after registration^11^. To initialise the registration between annotated histopathology and DTI-MRI, multiple control points were placed on the MR image and histopathology image. The pathologist and radiation oncologist worked in conjunction to select a minimum of 7 control points based on histopathology landmarks described above. The co-registration method used the co-ordinates of the control points to compute a similarity transformation, by minimising the Euclidean distance between the selected control points.

The *ex vivo* MRI – histopathology co-registration results were then qualitatively validated by the pathologist and radiation oncologist working in conjunction. This involved visual assessment of alignment on co-registered *ex vivo* MRI – histopathology of (i) anatomic landmarks used to drive registration, (ii) additional tissue regions of interest contours, including tumour, desmoplasia, fibrous tissue and rectal wall layers, independent of those used to drive registration, and (iii) geometry of the tissue specimen.

### MRI analysis

Histopathology was the standard reference for analysis. Regions of interest for analysis on annotated histopathology slides were identified on the matching DTI slice by visual inspection. MR image and quantitative measurements of DTI were performed in Amira 6 (FEI Visualization Sciences Group, Mérignac Cedex, France). The rectal tissue specimen was contoured on the selected DTI slice for analysis to provide a rectal mask. For MRI analysis, regions of interest were placed on cancer, desmoplasia, fibrous tissue, mucosa, submucosa, and muscularis propria. All voxels within regions of interest were included for analysis. The A0, ADC and FA maps were generated from the DTI-MRI dataset and used to probe rectal cancer stromal heterogeneity. To completely determine the diffusion tensor, diffusion measurements along at least six non-collinear directions are required. A0 and ADC can both be acquired from a subset of directions but FA requires determination of the full diffusion tensor.

The signal intensity from the A0 images were obtained. ADC values were calculated using the formula ADC = (*D*_xx_ + *D*_yy_ + *D*_zz_)/3 where *D*_xx_, *D*_yy_ and *D*_zz_ are the diagonal elements of the diffusion tensor. The FA values were calculated using the formula

$$FA=\sqrt{\frac{3}{2}}\frac{\sqrt{{({\lambda }_{1}-\langle \lambda \rangle )}^{2}+{({\lambda }_{2}-\langle \lambda \rangle )}^{2}+{({\lambda }_{3}-\langle \lambda \rangle )}^{2}}\,}{\sqrt{({{\lambda }_{1}}^{2}+\,{{\lambda }_{2}}^{2}+\,{{\lambda }_{3}}^{2})}}$$

where the *λ*_1_, *λ*_2_, *λ*_3_, and 〈*λ*〉 are the diffusion eigenvalues in three orthogonal directions and their average value, respectively^12^. FA maps were generated on a voxel-by-voxel basis with FA = 0 indicating isotropic diffusion (disorganised) and FA = 1 indicating anisotropic diffusion (organised) and provide a greyscale image of variations in fractional anisotropy. Direction encoded colour FA maps that show anisotropy in different colours according to the direction of the major axis were also produced; the colours green, blue and red were assigned to three orthogonal orientations.

### Statistical analysis

A linear mixed-effects model using a random intercept to control for repeated measures on the same individual was used to explore the relationship between estimated mean A0, ADC, and FA and tissue regions of interest. Following determination that the mean A0, ADC, and FA for at least one pair of tissue regions differed significantly, Dunnett’s multiple comparisons^13^ with control (cancer) methods were used to determine which tissue regions (if any) differed compared to cancer. A 5% significance level was used throughout the paper. The fold difference in A0, ADC and FA between cancer and other tissue regions was calculated using the ratio of mean values. Fold difference provides a useful unitless interpretation of the differences allowing comparison between the MRI measurements. A number of packages within R version 3.5.1 (R Core Team 2018, Vienna, Austria) statistical software were used for all analyses.

## Results

### Patients

A total of 10 rectal tissue specimens were collected by the SWSLHD CONCERT Biobank for this study from 5 patients with a diagnosis of rectal adenocarcinoma undergoing surgery. Three patients were male, two were female. The age range was 56–89 years. All patients had upper rectal tumours. All patients underwent primary surgery; 4 patients had an anterior resection and 1 had a pelvic exenteration. No patients had neoadjuvant radiotherapy or chemotherapy. Nine rectal tissue specimens were analysed at 11.7 tesla. One normal rectal specimen which was analysed at 14 tesla was excluded.

### MRI – Histopathology co-registration

**Figure 1:**
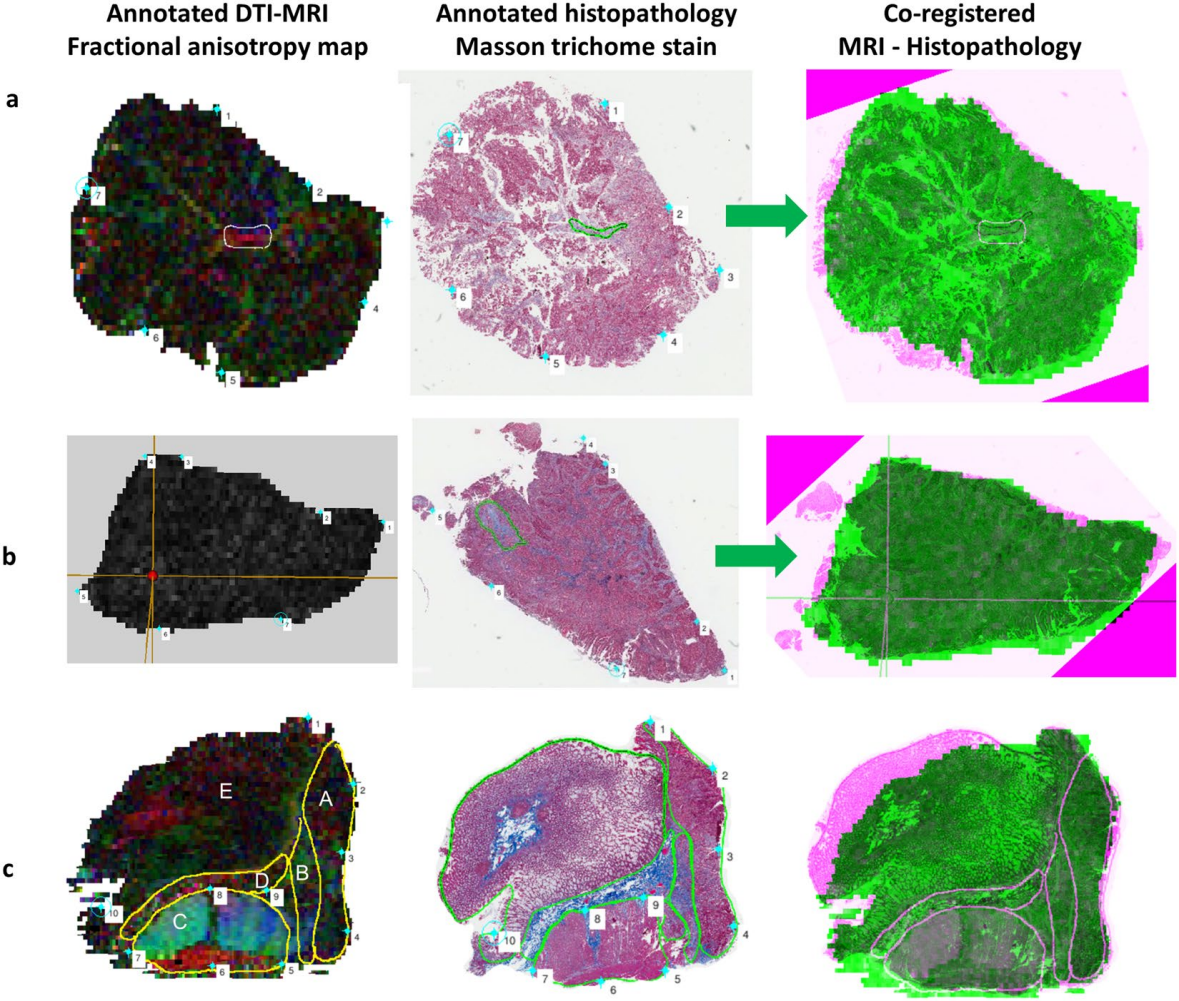
MRI – Histopathology co-registration process. Images for co-registration process performed using a co-registration method developed by Reynolds *et al*.^11^. The diffusion tensor imaging fractional anisotropy (FA) map (first column) was fused with the annotated histopathology (middle column). The MRI-histopathology co-registration image in shown in the last column. **(a)** A region of fibrous tissue was contoured on FA map and histopathology. Evaluation of the co-registration results showed good correlation between MRI and histopathology. **(b)** A region of demosplasia annotated on the FA map (cross-hairs) and histopathology matched well on the MRI-histopathology image. There was a small triangular area of tissue fragmentation that occurred during histopathology slicing and mounting onto the slide. **(c)** The contoured regions of interest on FA map and histopathology match well on the co-registered image. Annotated regions were A cancer, B desmoplasia, C muscularis propria, D fibrous tissue and E mucosa. Mucosa was flattened on histopathology mounting, resulting in some co-registration discrepancy in this region. The results show the value of MRI in assessing the true geometry of the tissue, within minimal distortion, allowing for MRI-histopathology correlation.

MR images and histopathology analysis showed that the tissue preparation method used in this study was able to maintain tissue integrity between MR scanning and histopathology. The MRI – histopathology co-registration results shown in Fig. 1 confirmed good correlation between annotated MRI and histopathology. Qualitative assessment of the co-registration results demonstrated good alignment of anatomic landmarks used to drive registration, additional annotated regions of interest and tissue geometry between *ex vivo* MRI and histopathology (Fig. 1). The results demonstrated good geometric preservation of the rectal specimens from MR analysis to histopathology analysis. The suspension of rectal specimens within the agarose gel allowed prevention of tissue folding during MR imaging. Our co-registration results showed there was a minimal amount of tissue fragmentation, which occurred at the step of histopathology slicing and mounting onto slides. The results show the value of MRI in being able to assess the true geometry of the specimen and avoid fragmentation and distortion, thereby allowing for direct correlation with histopathology analysis of the same tissue sample after MR imaging.

### Diffusion tensor imaging evaluation of rectal cancer stroma and adjacent normal rectum

**Figure 2:**
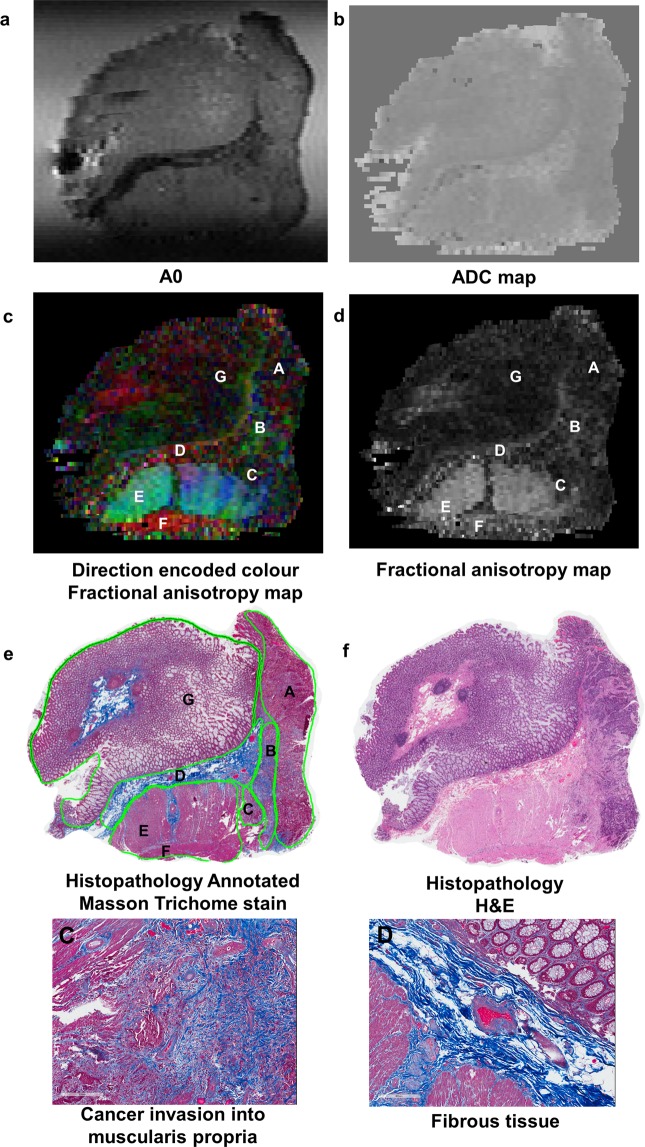
High field DTI-MRI and histopathology correlation results for rectal cancer tissue specimen 1. The DTI-MRI images shown are **(a)** A0, **(b)** ADC map, **(c)** direction encoded colour fractional anisotropy (FA) map, and **(d)** FA map. The corresponding histopathology is shown in **(e)** and **(f)**. The annotated regions on the diffusion tensor image and histopathology (including zoomed images) are: A cancer B desmoplasia C cancer invasion into muscularis propria D fibrous tissue E muscularis propria inner circular layer F muscularis propria outer circular layer and G mucosa. The A0 image was able to identify the band of fibrous tissue which had lower signal intensity. Cancer appeared hypointense on the ADC map, indicating restricted diffusion in cancer. The direction encoded colour FA map was the best MRI image for distinguishing the different tissue regions of interest; cancer and muscularis propria were most clearly distinguished on this image.

**Figure 3:**
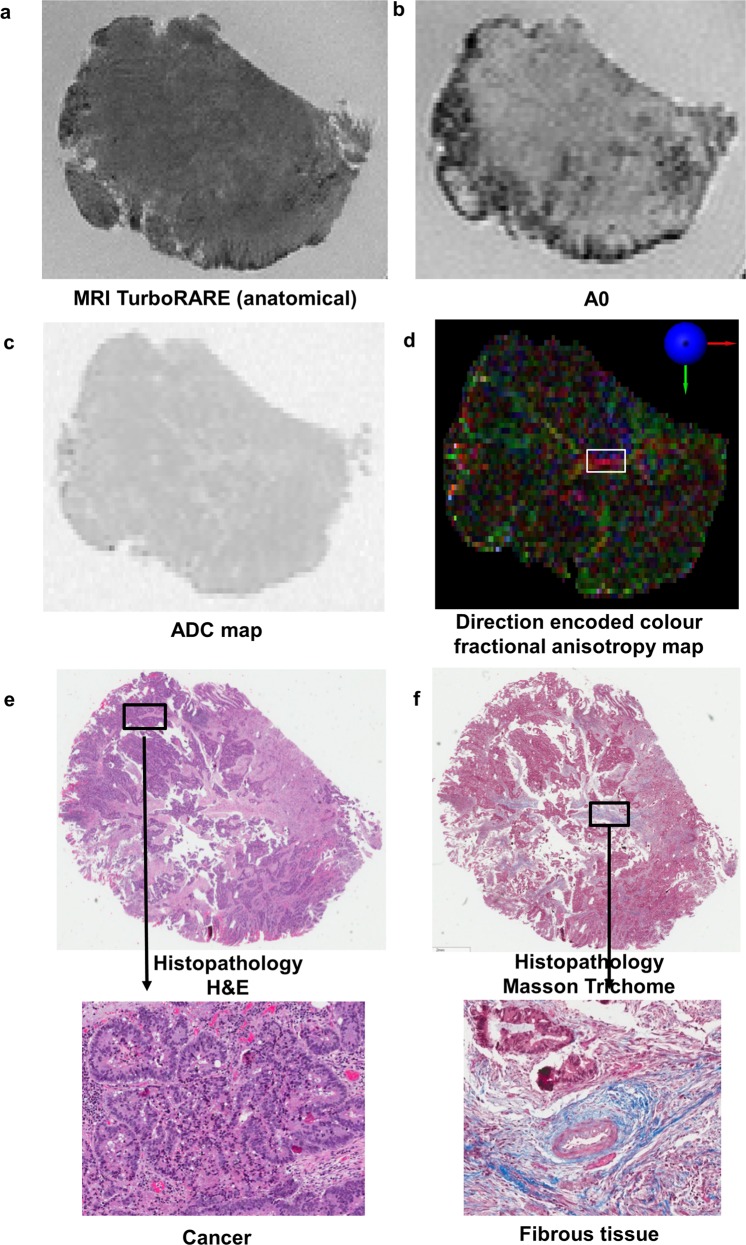
High field DTI-MRI and histopathology correlation results for rectal cancer tissue specimen 2. **(a)** An MRI TurboRARE image was used as the reference image to obtain the same slice on the DTI-MRI dataset as histopathology for analysis. The DTI-MRI images shown are **(b)** A0, **(c)** ADC map, and **(d)** direction encoded colour fractional anisotropy map. **(e)** Histopathology haematoxylin and eosin (H&E) stain with a region of cancer zoomed in. **(f)** Histopathology masson trichome stain with a region of mature fibrous tissue zoomed in. The direction encoded colour fractional anisotropy map was the best DTI-MRI derived image to identify fibrous tissue within the cancer specimen; fibrous tissue had brighter signal intensity and higher fractional anisotropy value than cancer.

**Figure 4:**
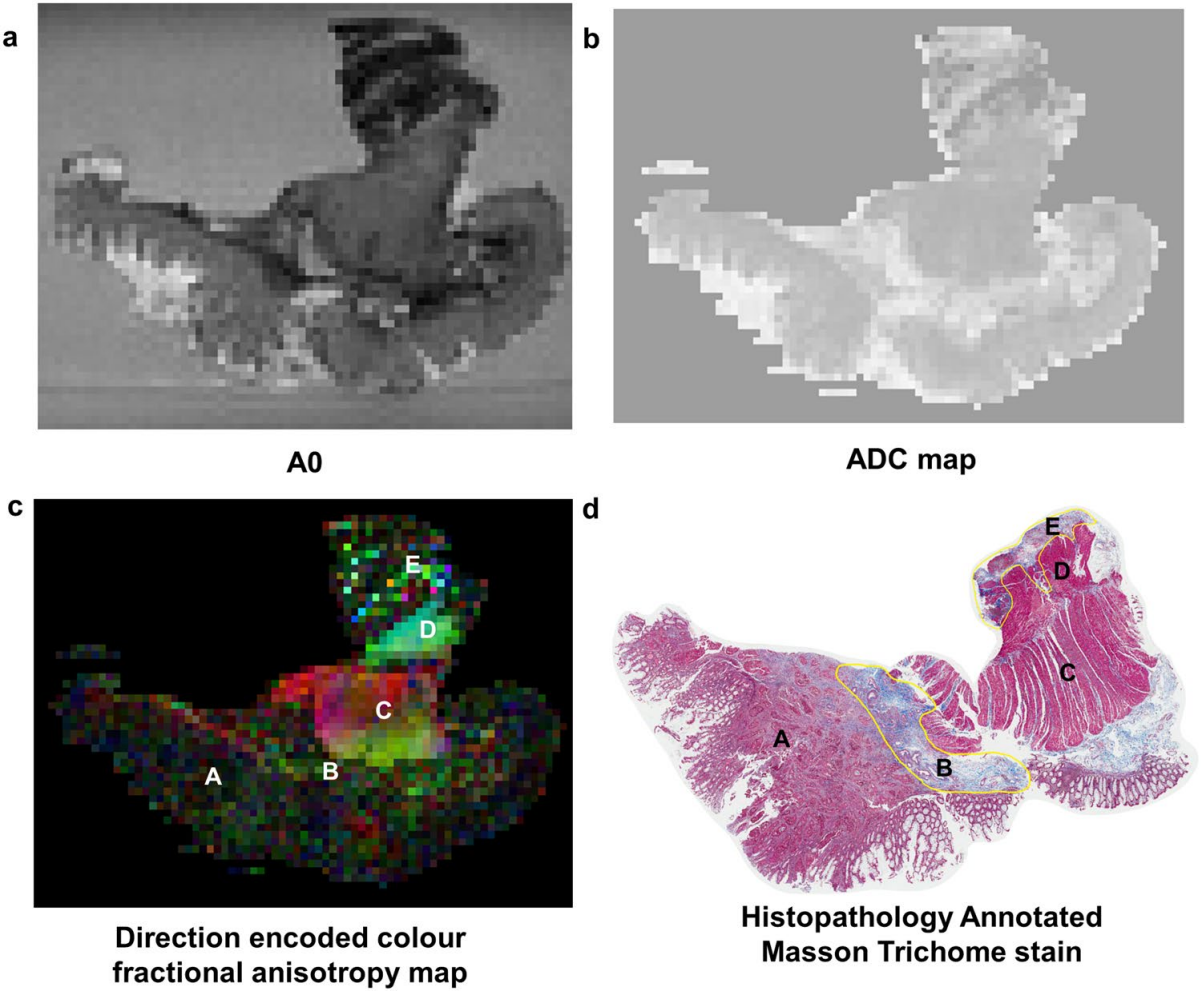
High field MRI and histopathology correlation results for rectal cancer tissue specimen 3. The MRI images shown are **(a)** A0, **(b)** ADC map, and **(c)** direction encoded colour fractional anisotropy (FA) map. The annotated regions are diffusion tensor image and histopathology are: A cancer B desmoplasia C muscularis propria inner circular layer D muscularis propria outer longitudinal layer and E heterogeneous regions of granulation tissue and inflammation. The direction encoded colour FA map was the best image to distinguish the different tissue regions of interest; muscularis propria was clearly distinguished from cancer on this image.

**Figure 5:**
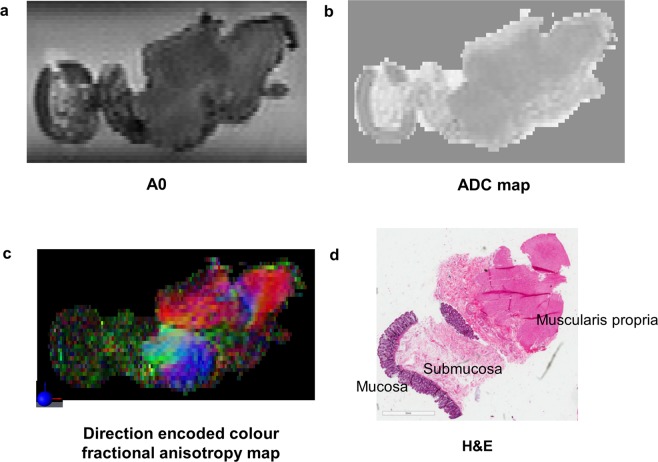
High field MRI and histopathology correlation for adjacent normal rectum tissue. The muscularis propria layer was most clearly identified on the direction encoded colour FA map.

Ultra-high resolution DTI-MRI was able to depict stromal heterogeneity in rectal cancer. Of the A0 images, ADC maps and diffusion encoded colour FA maps, the diffusion encoded colour FA maps provided the best contrast for depicting the different tissue regions of interest. The A0 images showed that cancer had a higher signal intensity compared to fibrous tissue (Fig. 2a). However, the differences between cancer and other tissue regions of interest were less obvious on qualitative evaluation of the A0 images. On the ADC maps it was difficult to differentiate the different tissue regions of interest on qualitative evaluation. The direction encoded colour FA maps exhibited relatively low signal intensity in regions of cancer, indicating relatively isotropic diffusion (Fig. 3). DTI-MRI allowed heterogeneity within the cancer stroma to be visualised, with regions of relatively high signal intensity corresponding to desmoplasia (Figs 2 and 4) or normal fibrous tissue (Figs 2 and 3) on direction encoded colour FA maps. Cancer invasion into muscularis propria (stage T2) was identifiable in the direction encoded colour FA map (Fig. 2). Muscularis propria was clearly distinguished from tumour, with muscularis propria having high signal intensity with highly anisotropic FA maps (Figs 2, 4 and 5). Muscularis propria, and its different muscular fibre orientations of the inner circular and outer longitudinal layers were able to be visualised on the direction encoded colour FA maps, and corresponded well with histopathology. Amongst the A0 images, ADC maps and direction encoded colour FA maps, muscularis propria was most clearly distinguished from cancer on the direction encoded colour FA maps.

### A0, ADC and FA values of rectal cancer and adjacent normal rectum

The DTI-MRI derived A0, ADC and FA mean estimate and standard error for rectal cancer and other tissue regions of interest are tabulated in Table 1. The A0 mean of cancer was significantly higher than all other tissue regions of interest (p < 0.001). The A0 image was most useful in discriminating between cancer and fibrous tissue (× 0.37 fold difference compared with cancer). The ADC mean of cancer was significantly lower than desmoplasia (p = 0.046), fibrous tissue (p = 0.026), mucosa (p < 0.001) and submucosa (p < 0.001), and significantly higher than muscularis propria (p < 0.001). The ADC mean was useful in discriminating between cancer and submucosa (× 1.82 fold increase compared with cancer). However, the fold differences in ADC for cancer and other tissue regions were very small (× 0.93–1.09). The FA mean of cancer was significantly lower than desmoplasia, fibrous tissue, submucosa and muscularis propria (p < 0.001). The FA mean of cancer was low (0.14) indicating near-isotropic diffusion and lack of stromal organisation. Desmoplasia and fibrous tissue had moderate mean FA values (0.31, and 0.34, respectively), indicating some degree of tissue organisation. Muscularis propria had high mean FA value (0.61), indicating highly organised and anisotropic diffusion. FA was most useful in discriminating between cancer and desmoplasia (× 2.15 fold increase compared with cancer), fibrous tissue (× 2.37 fold increase compared with cancer), and muscularis propria (× 4.25 fold increase compared with cancer).

**Table 1:** Estimated A0, apparent diffusion co-efficient (ADC), and fractional anisotropy (FA) means and standard errors (SE) (from the linear mixed effects model) for each tissue region of interest with p-values from Dunnett’s multiple comparison with cancer, and fold difference compared with cancer.

Tissue region of interest	A0		ADC		FA	
	Mean (SE) and p-value for comparison with cancer	Fold difference compared with cancer	Mean (SE) and p-value for comparison with cancer	Fold difference compared with cancer	Mean (SE) and p-value for comparison with cancer	Fold difference compared with cancer
Cancer	12405 (693)		0.002508 (0.00018)		0.1441 (0.011)	
Desmoplasia	8346 (766) p < 0.001	× 0.67	0.002736 (0.00019) p = 0.046	× 1.09	0.3096 (0.016) p < 0.001	× 2.15
Fibrous tissue	4646 (723) p < 0.001	× 0.37	0.002680 (0.00019) p = 0.026	× 1.07	0.3413 (0.013) p < 0.001	× 2.37
Mucosa	10543 (708) p < 0.001	× 0.85	0.002751 (0.00018) p < 0.001	× 1.09	0.1389 (0.012) p = 0.956	× 0.96
Submucosa	9612 (712) p < 0.001	× 0.77	0.004567 (0.00018) p < 0.001	× 1.82	0.1849 (0.012) p < 0.001	× 1.28
Muscularis propria	9896 (692) p < 0.001	× 0.80	0.002329 (0.00018) p < 0.001	× 0.93	0.6127 (0.011) p < 0.0001	× 4.25

**Figure 6:**
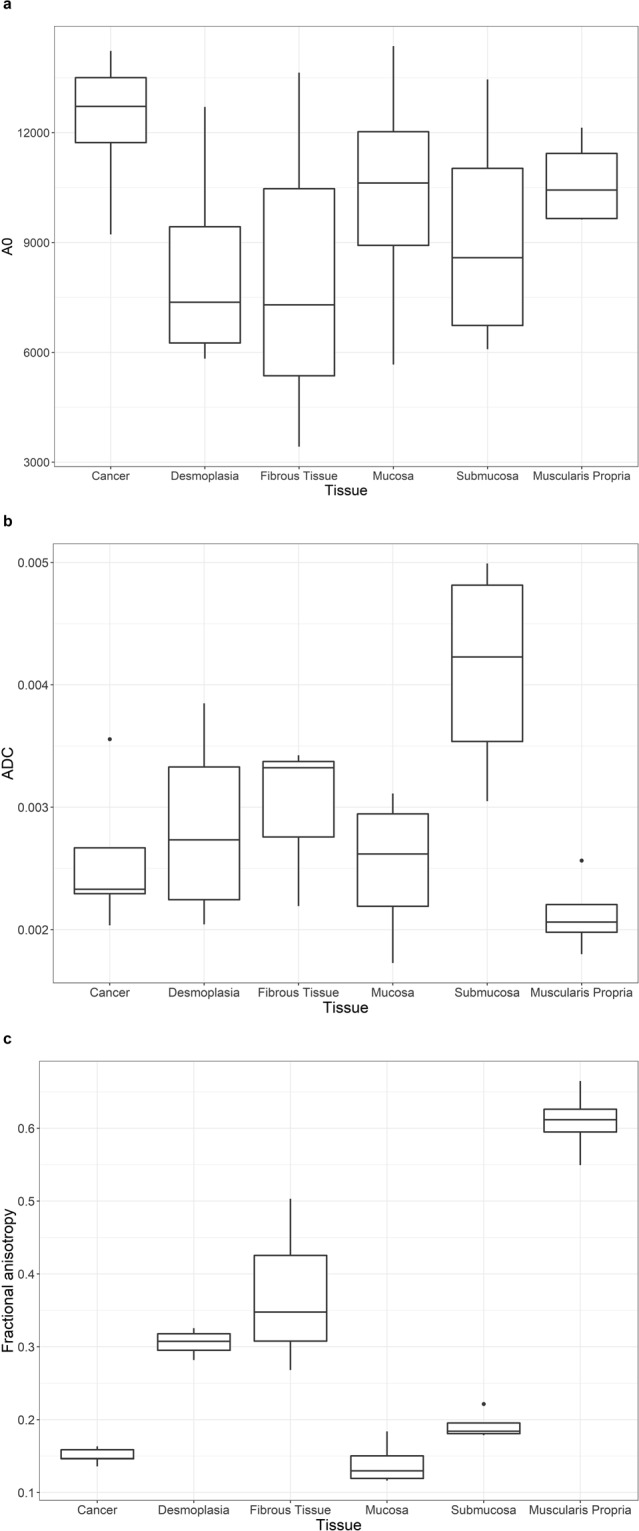
Box plots of (**a**) A0, (**b**) apparent diffusion co-efficient (ADC) and (**c**) fractional anisotropy (FA) values for each tissue region of interest for all patients. The boxes represent the 25^th^ to 75^th^ percentile (interquartile range), the lines within the box represent the median values and the whiskers show the range of values. The dots are outliers. The box plots showed that FA had the clearest contrast between cancer and desmoplasia, fibrous tissue and muscularis propria.

Figure 6 shows A0, ADC and FA box plots summaries for each tissue region of interest. The box plots summarise the upper and lower quartiles, and median values. Of the A0, ADC and FA box plots, FA had the greatest separation of median values and interquartile ranges between cancer and desmoplasia, fibrous tissue and muscularis propria. The box plots show that FA discriminates the most between cancer and desmoplasia, fibrous tissue, or muscularis propria.

## Discussion

This exploratory MRI – histopathology correlative study demonstrated the ability of DTI-MRI to assess rectal cancer stromal heterogeneity and tumour extent *ex vivo*. The DTI-MRI derived A0, ADC and FA values were able to differentiate between cancer and other tissue regions (desmoplasia, fibrous tissue, mucosa, submucosa and muscularis propria). The A0 images were useful in discriminating between cancer and fibrous tissue. The differences in ADC values between cancer and other tissue regions were very small. The direction encoded colour FA maps and FA values provided the best discrimination between cancer and regions of desmoplasia, fibrous tissue and muscularis propria. DTI revealed that tumour is disorganised and consequently has relatively isotropic diffusion (low FA). In contrast, desmoplasia and normal fibrous tissue have moderate stromal organisation and significantly higher FA than cancer. DTI was also useful in assessing the depth of tumour invasion into the rectal wall. DTI enabled clear differentiation of tumour from muscularis propria which was highly organised and anisotropic, allowing for detection of tumour infiltration into muscularis propria. This finding could be particularly useful in the differentiation between stage T1 and T2 tumours. This study has shown that FA constitutes a potential novel MRI biomarker of rectal cancer stromal organisation and infiltration *ex vivo*.

For each of the specimens three derived images were obtained from the DTI experiments, these being the A0, FA and ADC. Of these, the ADC and FA are derived from the diffusion tensor and are theoretically instrumentation independent. The A0 image is the intensity image when the diffusion gradients are zero. Its voxels are weighted by *T*_1_, *T*_2_ and proton density and its contrast is more dependent on the instrument and parameters. Thus the ADC and FA results are more suited to clinical translation as the contrast between tissue types should remain similar across instrumentation.

Our MRI – histopathology co-registration results demonstrated good correlation between MRI and histopathology. This study demonstrated the value of MRI in assessing the true geometry of tissue with little deformation. Tissue preparation and MRI scanning in this study resulted in no tissue destruction between imaging and histopathology, allowing for direct correlation with ‘ground-truth’ histopathology for analysis. The method of tissue fixation and suspension in agarose gel ensured preservation of tissue geometry and orientation from MR imaging to histopathology slicing resulting in a good match between MRI and histopathology. This study used an *ex vivo* MRI – histopathology co-registration framework developed and validated by Reynolds *et al*.^11^. The quantitative validation of the co-registration method by Reynolds *et al*. demonstrated a mean distance of 0.57 mm (range 0.06–1.99 mm) between the control points after registration. Various other MRI – histopathology correlation methods have been used in previous studies. Yamada *et al*. examined oesophageal and gastric cancer tissue *ex vivo* using DTI-MRI at 7 tesla^14,15^. They performed a qualitative visual correlation between MRI and histopathology, without formal co-registration, in their analysis. A technique for co-registration of *in vivo* MRI with histopathology in rectal cancer has been developed by Antunes *et al*.^16^. Antunes *et al*. imaged *in vivo* at 3 tesla. For *in vivo* imaging and histopathology co-registration, there are additional factors leading to tissue deformation that need to be considered. Rectal peristalsis *in vivo*, surgical removal of rectum and tissue fixation can lead to tissue deformation, shrinkage and distortion of tissue geometry between *in vivo* imaging and histopathology. A deformable registration process was used by Antunes *et al*. to overcome this. The spatial alignment strategies used in this study were similar to that used by Antunes *et al*., with both studies visually identifying landmarks on histopathology corresponding to edges or curvatures along the rectal wall to drive the MRI – histopathology co-registration procedure. Antunes *et al*. quantitatively validated their co-registration results by using additional independent landmarks. Their quantitative validation demonstrated excellent co-registration with overall deviation of 1.50+/−0.63 mm between *in vivo* MRI and histopathology. The present study performed a qualitative validation of the co-registration. A quantitative validation of the co-registration results was not performed, as the co-registration method had previously been validated. The present study was performed at ultra-high field (11.7 tesla). A huge advantage of ultra-high field imaging is that the high resolution images allows excellent visualisation of tissue regions such as rectal wall layers and fibrous tissue, otherwise not seen at low field, to then validate co-registration results. Ultimately, the ‘microscopic’ resolution of images at ultra-high field allows visual confirmation of the co-registration results.

MRI plays an important role in the primary staging of rectal cancer, and is the staging imaging of choice. Despite the important role of conventional MRI for staging rectal cancer, there are some limitations. Conventional high spatial resolution *T*_2_-weighted MRI has moderate reproducible accuracy in the prediction of tumour stage of rectal cancer, with accuracy of 67% and 83% by 2 independent observers in a study by Beets-Tan *et al*.^17^ A study by Brown *et al*. found that the majority of disagreements between thin-slice *T*_2_-weighted MRI and histopathology were between T1 and T2 tumours, and between T2 and T3 tumours^18^. Conventional MRI is poor at separating stage T1 from stage T2 rectal tumours, making it inadequate in the selection of patients for local excision^4,5,18,19^. A desmoplastic reaction in rectal cancer can also pose challenges in accurate staging of rectal cancer, as desmoplastic stranding can appear similar to tumour on MRI, resulting in overestimation of tumour extent. The presence of fibrous tissue can also lead to staging difficulty as this has similar appearance to tumour on conventional *T*_2_-weighted MRI^20^. This study has shown that DTI-MRI was clearly able to differentiate cancer from muscularis propria. DTI-MRI was able to visualise tumour invasion into muscularis propria, with disruption of the muscularis propria at the invasive tumour front. In addition, DTI-MRI was able to assess cancer stromal heterogeneity and was able to distinguish desmoplasia or normal fibrous tissue, with these regions having significantly higher FA than cancer. FA was able to characterise fibril anisotropy as a probe of stromal microenvironment.

This study has also shown the power of quantitative analysis of DTI-MRI in detecting differences in A0, ADC and FA between cancer and other tissue regions that were not obvious on qualitative review of the images. The FA value for cancer was significantly different compared with all other tissue regions, except mucosa. DTI-MRI may add value in more accurately defining tumour extent in rectal cancer which would assist with surgical planning and warrants further investigation. Yamada *et al*. have examined DTI-MRI at 7 tesla in oesophageal and gastric cancers *ex vivo* and found that DTI was a feasible means of evaluating mural depth of invasion^14,15,21^. To the best of our knowledge, there are no other published studies examining the role of DTI-MRI in rectal cancer.

This study was performed on an ultra-high field 11.7 tesla MRI, with a magnet bore size of 89 mm. An advantage of higher field is the approximately linear increase in signal, resulting in the potential to acquire data with improved spatial resolution. An increase in signal-to-noise ratio is also important for functional imaging techniques. Most clinical MRI systems are now available at both 1.5 and 3 tesla, and it is anticipated that over the next decade the market share of clinical MRI systems will change toward higher field strengths^22^. Human MRI scanners at 7 tesla have been installed in recent years^23,24^. Preliminary results from human brain MRI studies at 9.4 tesla have indicated that safe and successful human imaging is feasible at even higher field strengths^25^. Ongoing work into increasing the field strengths of clinical MRI scanners and the adoption of clinical 7 tesla MRI scanners, should facilitate the translation of this DTI-MRI protocol to clinical usage.

This study focused on the potential of DTI-MRI in the differentiation of stromal heterogeneity and primary staging of rectal cancer. Our results demonstrated the feasibility of DTI-MRI in distinguishing fibrosis from tumour. We propose that this finding could be useful in the assessment of chemoradiotherapy response and should be investigated in this setting. In patients who have received neoadjuvant chemoradiotherapy, the role of diffusion weighted imaging (DWI) in the clinical assessment of response is emerging^26–32^. However, the differentiation of radiotherapy-induced fibrosis from residual tumour on *T*_2_-weighted and DWI-MRI remains a challenge^33^. Radiotherapy-induced fibrosis can result in restricted diffusion that is detectable in DWI, mimicking the appearance of residual tumour and making it difficult to correctly identify pathologic complete responders to chemoradiotherapy. Jang *et al*. found that 42% of patients with pathologic complete response had restricted diffusion on DWI-MRI, and that radiotherapy-induced fibrosis was a significant predictor of diffusion restriction. The potential of DTI-MRI has not yet been investigated in patients who have received neoadjuvant radiotherapy; whether DTI-MRI can add value to current clinical MRI techniques in distinguishing residual tumour from fibrosis and contribute to radiotherapy response assessment should be investigated.

There were a number of limitations in this study. Firstly, the sample size in this study was small due to the limited number of rectal cancer patients participating in CONCERT Biobank. Secondly, this study did not compare DTI-MRI with standard *T*_2_-weighted MRI. A future study with a larger sample size would enable assessment of the accuracy (sensitivity and specificity) of DTI-MRI in defining tumour extent in rectal cancer compared with conventional *T*_2_-weighted MRI. Thirdly, the scan time was considerably long. Reducing the number of *b*-values and utilising image acceleration would substantially reduce the scan time. We are currently working on further DTI-MRI experiments at 7 and 9.4 tesla fields to facilitate clinical translation of our findings.

In conclusion, this exploratory MRI-histopathology correlative study demonstrated the ability of DTI-MRI to examine rectal cancer stromal heterogeneity and tumour extent at high field *ex vivo*. DTI-MRI derived A0, ADC and FA values made it possible to probe rectal cancer stromal heterogeneity. FA provided the best discrimination between cancer and desmoplasia, fibrous tissue and muscularis propria. This study has shown that FA is a potential novel biomarker of rectal cancer stromal organisation and tumour infiltration, *ex vivo*. DTI-MRI at 11.7 tesla was able to differentiate tumour regions from desmoplasia or fibrous tissue *ex vivo*; cancer had relatively isotropic diffusion, whereas regions of desmoplasia or fibrous tissue had anisotropic diffusion with higher FA than cancer. DTI-MRI was also useful in assessing extent of tumour infiltration into the rectal wall. DTI was able to identify cancer invasion into muscularis propria, which was highly anisotropic and clearly able to be distinguished from tumour. Thus, DTI-MRI may provide clear characterisation of tumour stromal heterogeneity and accurate delineation of tumour extent in rectal cancer and warrants further investigation.
